# Flame-Retardant and Smoke-Suppressant Flexible Polyurethane Foams Based on Phosphorus-Containing Polyester Diols and Expandable Graphite

**DOI:** 10.3390/polym15051284

**Published:** 2023-03-03

**Authors:** Hongkun Wang, Qiang Liu, Hui Li, Hao Zhang, Shouke Yan

**Affiliations:** Key Laboratory of Rubber-Plastics, Ministry of Education/Shandong Provincial Key Laboratory of Rubber Plastics, Qingdao University of Science & Technology, Qingdao 266042, China

**Keywords:** flexible polyurethane foams, polyester diols, expandable graphite, flame retardant

## Abstract

A liquid-phosphorus-containing polyester diol, PPE, was prepared via condensation polymerization using commercial reactive flame retardant 9,10-dihydro-10-[2,3-di(hydroxycarbonyl)propyl]-10-phospha-phenanthrene-10-oxide, adipic acid, ethylene glycol, and 1,4-butanediol. PPE and/or expandable graphite (EG) were then incorporated into phosphorus-containing flame-retardant polyester-based flexible polyurethane foams (P-FPUFs). The structure and properties of the resultant P-FPUFs were characterized using scanning electron microscopy tensile measurements, limiting oxygen index (LOI), vertical burning tests, cone calorimeter tests, thermogravimetric analysis coupled with Fourier-transform infrared spectroscopy, X-ray photoelectron spectroscopy, and Raman spectroscopy. Unlike the FPUF prepared using regular polyester polyol (R-FPUF), PPE increased the flexibility and elongation at break of the resultant forms. More importantly, the peak heat release rate (PHRR) and total heat release (THR) of P-FPUF were reduced by 18.6% and 16.3%, respectively, via gas-phase-dominated flame-retardant mechanisms, compared with those of R-FPUF. The addition of EG further reduced the peak smoke production release (PSR) and total smoke production (TSP) of the resultant FPUFs while increasing the LOI and char formation. Interestingly, it was observed that EG noticeably improved the residual quantity of phosphorus in the char residue. When the EG loading was 15 phr, the resulting FPUF (P-FPUF/15EG) attained a high LOI value (29.2%) and exhibited good anti-dripping performance. Meanwhile, the PHRR, THR, and TSP of P-FPUF/15EG were significantly decreased by 82.7%, 40.3%, and 83.4%, respectively, compared with those of P-FPUF. This superior flame-retardant performance can be attributed to the combination of the bi-phase flame-retardant behavior of PPE and condensed-phase flame-retardant characteristics of EG.

## 1. Introduction

Flexible polyurethane foams (FPUFs), which are used as cushioning materials in furniture, bedding, automobile carpets, etc., account for approximately 40% of the market share of polyurethane materials. However, FPUFs are highly flammable because of their chemical nature, high air permeability, and large surface area [1,2,3,4,5,6,7,8,9,10]. Therefore, the synthesis of flame-retardant FPUFs is of great importance [11,12,13,14,15,16].

It was well-known that flame retardants can be physically coated onto or incorporated into the matrix material to create fire-resistant polyurethanes [17,18,19,20,21,22,23,24,25,26,27,28,29,30,31,32,33,34,35,36,37,38,39,40]. However, several problems, including weak compatibility, leaching, and poor mechanical properties, have been observed for some liquid-based flame-retardant additives [41]. Solid flame-retardant expandable graphite (EG), with a flake-like graphite structure, is known to reduce the smoke release, melt-drip, and flammability of polyurethanes via the condensed-phase mechanism of fire retardancy [42,43,44,45,46]. On the other hand, a number of reactive flame retardants containing phosphorus and/or nitrogen groups have been developed and incorporated into the polymer matrix [47,48,49,50]. Unfortunately, many of the phosphorus-containing flame retardants incorporated into polyurethane cause the deterioration of the smoke release and melt-drip resistance of the polymer during combustion [41,42,49], resulting in serious suffocation and scalding. One strategy for overcoming this is to combine EG and the reactive flame retardant to provide additional or synergistic effects [49,51,52,53,54,55,56,57,58,59]. Rao et al. [49] designed and synthesized flame-retardant and smoke-suppressant FPUFs based on EG and the liquid-phosphorus-containing polyol (PDEO). The vertical burning test revealed that the FPUF containing 5 phr PDEO and 10 phr EG extinguished quickly without dripping and kept the original shape after removing the igniter [49]. Recently, Ma et al. [57] incorporated EG into a novel phosphine-oxide-containing hyperbranched polyol and observed a significantly reduced release of contaminative smoke as well as carbon and nitrogen oxides from fires.

9,10-Dihydro-10[2,3-di(hydroxycarbonyl)propyl]10-phosphaphenanthrene-10-oxide (DDP) has been shown as an effective reactive phosphorus-containing monomer in the synthesis of flame-retardant polyester diols. Meanwhile, these polyester diols have been employed in the synthesis of flame-retardant polyurethane elastomers and rigid polyurethane foams [51,60]. Polyester-based FPUFs are widely used in luggage, footwear, textile and oil tank filling industries due to their high resilience, high tensile strength and tear strength, as well as oil resistance. The aim of this work was to synthesize flame-retardant, low-smoke-releasing, and non-melt-dripping polyester-based FPUFs by incorporating a liquid-phosphorus-containing flame-retardant polyester (PPE) diol synthetized via condensation polymerization with DDP, adipic acid (AA), ethylene glycol (EG), and 1,4-butanediol (1,4-BDO). As expected, phosphorus-containing flame-retardant FPUFs (P-FPUF), with improved limiting oxygen index (LOI) values and residual char yields, were successfully obtained, but high smoke releases during the cone calorimetry test were observed. The incorporation of EG into the P-FPUFs decreased the smoke and total heat releases via the mechanism of flame retardancy. The flame-retardant characteristics were studied carefully to ultimately determine the flame retardancy mechanism.

## 2. Experiment

### 2.1. Materials

Materials used in this study are given in the Supplementary Material.

### 2.2. Synthesis of PPE

**Scheme 1:**
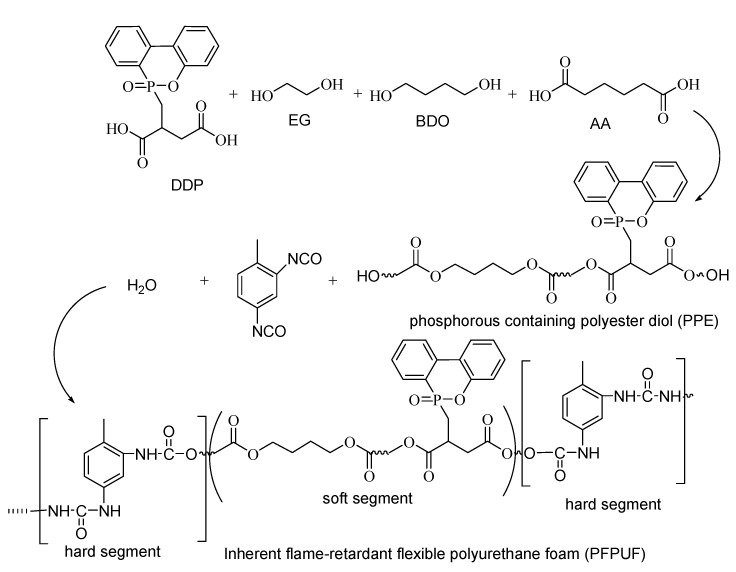
Synthetic route of PPE and PFPUF.

The synthetic route of the PPE is summarized in Scheme 1. DDP (0.8 mol, 276.94 g), adipic acid (AA; 13.20 mol, 1926.19 g), 1,4-BDO (9.10 mol, 819.27 g), ethylene glycol (EG; 9.10 mol, 546.18 g), and catalyst (tetrabutyl titanate, TBT; 60 ppm) were cast into a 5000 mL customized reactor equipped with nitrogen gas passed in. The reaction was heated from room temperature to 140 °C, the stirring continued to run during this period, and the reaction was continued at 140 °C for 3 h. The temperature was then slowly raised to 220 °C in 4 h to complete the esterification process, and the byproducts and unreacted glycol were pumped out until the acid value was less than 1 mg KOH/g. The viscosity of PPE was 12,500 cps at 25 °C.

### 2.3. Synthesis of FPUFs

The FPUFs were prepared by the one-pot and free-rise method. The formulations of FPUFs are listed in Table S1. Polyester diols, catalysts, silicone oil, distilled water, and expandable graphite (EG) were mixed under vigorous stir. TDI was subsequently added to the homogeneous mixture and stirred for 10 s. The foams were cured at 80 °C for 6 h. Synthesis of FPUFs is shown in Scheme 1.

### 2.4. Characterization

Detailed information is given in the Supplementary Material.

## 3. Results and Discussion

### 3.1. Characterization of PPE

**Figure 1:**
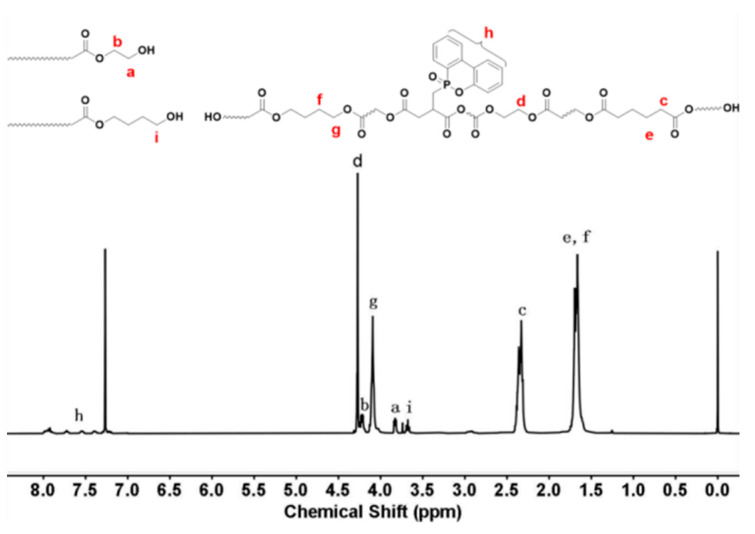
^1^H NMR spectrum of PPE.

The chemical structure of PPE was characterized by proton nuclear magnetic resonance (1H NMR) (Bruker spectrometer (500 MHz), Germany). The 1H NMR spectrum of PPE is illustrated in Figure 1. The signal peaks at 1.67 (b) and 2.33 (a) ppm were assigned to the methylene in AA; the peaks at 1.67 (e), 3.66 (g), and 4.10 (d) ppm were attributed to methylene in 1,4-BDO; and the peaks at 3.81 (f), 4.21 (h), and 4.28 (c) ppm were ascribed to methylene in EG. The chemical shifts (i) in the range of 7.28−7.98 ppm were clearly assigned to the aromatic protons in DDP [51,60]. The molecular weight of PPE was measured by gel permeation chromatography (GPC) (SHIMADZU EcoSEC8320, Japan). As shown in Figure S1, the weight-average molecular weight (Mw) and molecular weight distribution (Mw/Mn) were 2446 g/mol and 1.65 based on polystyrene standard, respectively, indicating that the PPE had a relatively narrow molecular weight distribution.

### 3.2. Study of Morphologies and Mechanical Properties

**Figure 2:**
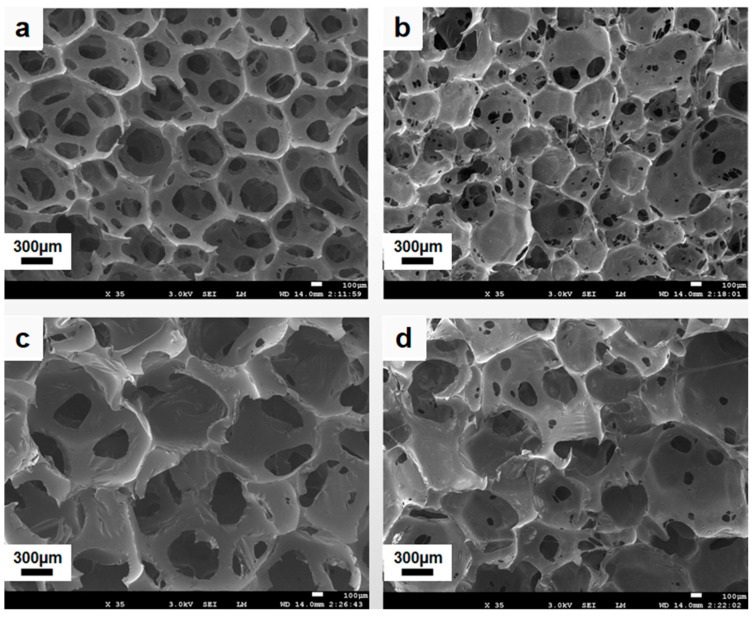
SEM images for cross-sections of pure FPUF (**a**), FPUF/10EG (**b**), FPUF/P (**c**), and P-FPUF/10 EG (**d**), ×35.

Flame-retardant FPUFs were synthesized by incorporating EG via a facile one-pot route. The densities of the resultant flame-retardant EG-containing FPUFs are in the range of 45−91 kg/m^3^ (Table S1). The surface morphologies of the FPUFs were characterized using SEM (Manufacturer, city, state abbreviation, country). As shown in Figure 2 and Figure S2, the FPUFs prepared with RPE (R-FPUFs) and FRPE (P-FPUFs) have open cellular structures, and the former has relatively small dimensions. EG can affect the nucleation and growth of bubbles, which increases the foam density [49]. Figure 2b,d show that the introduction of EG particles has an apparent effect on cell structure; the crude cross-sections and collapse of the cellular structures are observed.

### 3.3. Flame Retardancy

**Figure 3:**
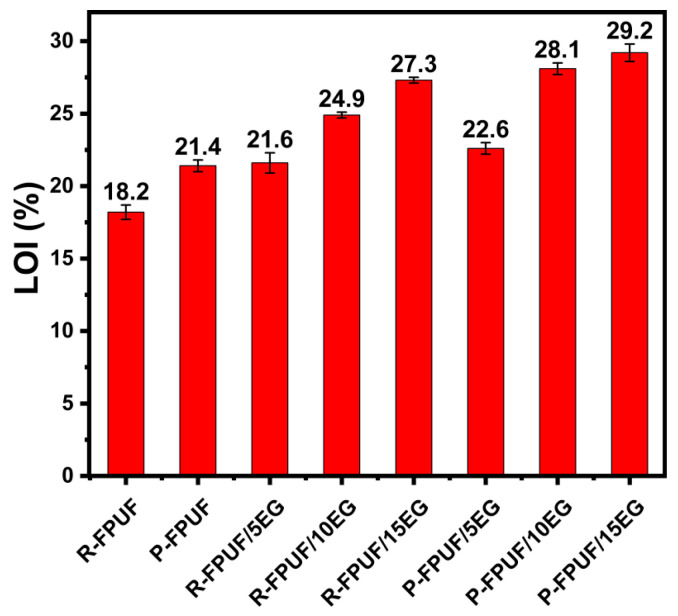
LOI bar graph of R-FPUF and flame-retardant FPUFs.

R-FPUF has a poor flame retardancy as indicated by the low LOI value of 18%. As shown in Figure 3, the incorporation of phosphorus and EG increases the LOI. The LOI value of P-FPUF is 21%, which is higher than that of R-FPUF (18%). It is worth noting that the LOI value of R-FPUF/10EG is 25%, whereas the LOI value of P-FPUF/10EG is 28%, suggesting that there is an additional flame-retardant effect due to the presence of EG [54,56].

**Figure 4:**
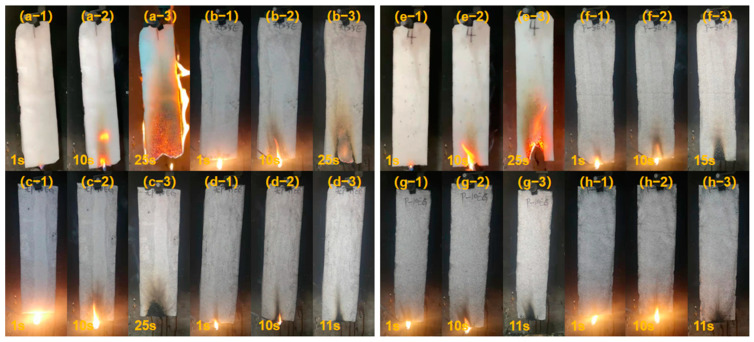
Digital photos of R-FPUF (**a–1**~**a–3**), R-FPUF/5EG (**b–1**~**b–3**), R-FPUF/10EG (**c–1**~**c–3**), R-FPUF/15EG (**d–1**~**d–3**), P-FPUF (**e–1**~**e–3**), P-FPUF/5EG (**f–1**~**f–3**), P-FPUF/10EG (**g–1**~**g–3**), P-FPUF/15EG (**h–1**~**h–3**)at different ignition time.

The results of the vertical burning tests for the flame-retardant foams are shown in Figure 4, and the tested parameters are listed in Table S1 and S2. As shown in Figure 4a, R-FPUF burns out rapidly with severe dripping after removing the pilot flame. As for P-FPUF, the spreading speed of the fire (Figure 4e) is lower, but melt-dripping still occurs, and the foam burns out after removing the igniter. The incorporation of EG significantly decreases the flame spreading rate and prevents melt-dripping. P-FPUF/5EG exhibits excellent self-extinguishing behavior and no melt-dripping and thereby passes the vertical burning test (Table 1). Most notably, R-FPUF/10EG failed to pass the vertical burning test. This again indicates a better flame-retardant performance of FRPE than RPE in the case of the same dosage of EG.

### 3.4. Fire Behavior

**Figure 5:**
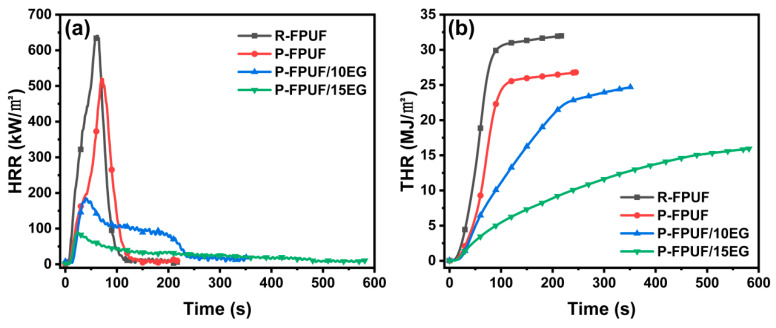
Heat release rate (**a**) and total heat release (**b**) curves for FPUFs under an external heat flux of 25 kW/m^2^.

The relevant parameters, including time to ignition (TTI), total heat release (THR), peak heat release rate (PHRR), time to peak heat release rate (TPHRR), fire growth rate index (FIGRA), total smoke production (TSP), average effective heat of combustion (AEHC) from ignition to flame out, carbon monoxide production (COP), and char yields (CY), are shown in Table 2 and Figure 5. The TTI, THR, and PHRR values of R-FPUF are 9 s, 32.0 MJ/m^2^, and 640 kW/m^2^, respectively, whereas the corresponding values for P-FPUF are 12 s, 26.8 MJ/m^2^, and 521 kW/m^2^, indicating that the phosphorus-containing FRPE is highly effective in improving the flame-retardant property of the FPUF. When compared with R-RPUF, the char residue of P-FPUF after cone calorimeter (CC) testing increases owing to the introduction of FRPE, which is consistent with the Thermogravimetric analysis (TGA) results.

As it can be seen in Table 2 and Figure 5, the incorporation of 10 phr EG decreases the THR and PHRR values significantly. This is because EG presents carbon layers in the shape of “worms”, forming a thermally insulating barrier. P-RPUF/10EG has a TTI of 19 s, which is longer than the 12 s of the control sample P-RPUF. Simultaneously, the PHRR and THR of P-RPUF/10EG reduced by 65% and 7.8%, respectively, when compared with P-RPUF. These results support the effect caused by the presence of phosphorus and EG.

**Figure 6:**
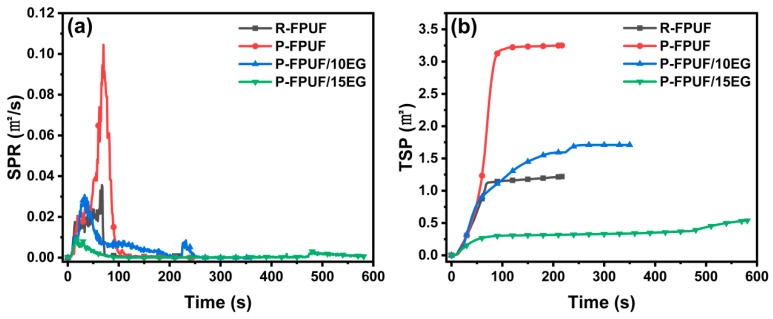
Smoke production rate (**a**) and total smoke production (**b**) for FPUFs.

**Figure 7:**
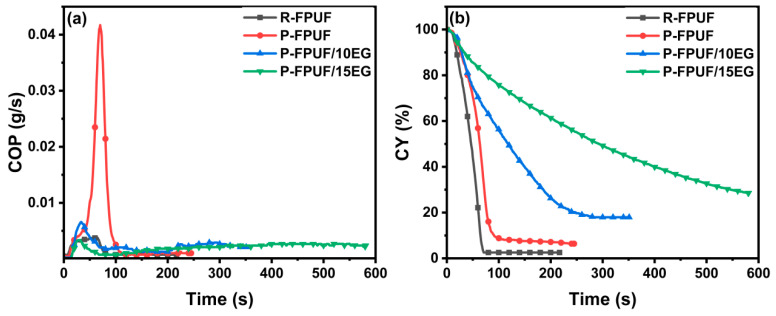
Carbon monoxide production (**a**) and char yields (**b**) curves for FPUFs.

As important parameters for evaluating a flame-retardant material, lower PSPR (peak of smoke production rate) and TSP values represent a lower risk of choking. As shown in Figure 6a, by comparing the PSPR values of R-FPUF (0.036 m^2^/s) and P-FPUF (0.104 m^2^/s), the incorporation of phosphorus increases the smoke release and carbon monoxide yield (COY), which can be attributed to the classical gas-phase flame-retardant mechanism of phosphorus-containing compounds during combustion [49,57,60,61]. As expected, with the incorporation of EG, the PSPR and SPR values of all FPUFs decrease significantly. This is mainly due to the thick carbonaceous shield resulting from the expansion of EG that can trap substantial amounts of gases and provide great charring ability [43,44,45,46,49,57]. It should be noted that the P-FPUF/10EG sample still exhibits higher PSPR and TSP values of 0.031 m^2^/s and 1.71 m^2^. Thus, 15 phr EG was further incorporated into F-FPUF. As shown in Figure 7 and Table 2, the PSRR and TSP of the resulting P-RPUF/15EG sample are reduced by 82.7% and 40.3%, respectively, when compared with P-RPUF, which became lower than that of the P-RPUF sample. However, the incorporation of 15 phr EG shortens the TTI of R-FPUF from 19 to 7 s, indicating an easier ignition. This can be explained by the accumulation of expanded EG in the foam matrix, which exposes the underlying materials to the flame [44,45,46].

The AEHC assesses the efficiency of a flame retardant in terms of flame inhibition and gas-phase activity [58,59,60]. With the incorporation of EG, all the AEHC values increase considerably when compared with those of the FPUFs without EG. Thus, it can be concluded that the flame retardancy of EG occurs in the condensed phase. The fire growth rate index (FIGRA, a deduced parameter which equals the maximum of HRR/time) indicates the deflagration of a material [60,61]. Fire safety was assessed through the fire growth rate index (FIGRA, the maximum ratio HRR (t)/t)). The lower the FIGRA value, the higher the fire safety. A clear decreasing trend is observed with increasing EG dosage, indicating an increase in fire safety when using the resultant FPUFs.

It should be noted that the incorporation of EG does not decrease the COY values of most foams. As displayed in Table 2, the incomplete burning is mainly due to gas-phase flame-retardant properties of phosphorus [47,49,60], which results in the slow and continuous release of CO, leading to high COY values.

### 3.5. Thermal Degradation of FPUFs

**Figure 8:**
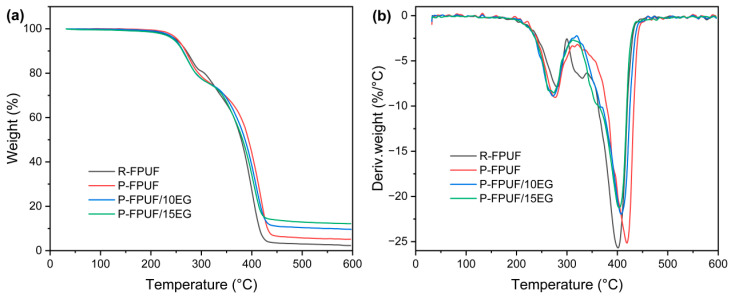
TGA (**a**) and derivative thermogravimetry (DTG) (**b**) curves for FPUFs at a heating rate of 20 °C/min in N₂.

TG analysis was performed to investigate the thermal stability and charring ability of the resultant foams. As can be seen in Figure 8, all the FPUFs undergo a two-step decomposition. The first weight loss is attributed to the breakage of the bonds in the TDI-derived moieties, while the second weight loss can be ascribed to the degradation of the soft segments [7,62]. The corresponding temperatures of maximum weight loss rate (T_max_, denoted as the differential thermogravimetry peak maximum), maximum weight loss rate (RT_max_), and residual weight at 600 °C (C_600_) are listed in Table 3. The lower T_max1_ and RT_max1_ of P-FPUFs indicate that the incorporation of the phosphorus-containing segment of DDP accelerates the first degradation step [60,61]. In contrast, the T_max2_ of P-FPUFs increases by approximately 20 °C when compared with that of R-RPUF, as also seen in previous studies [41,47,49,60]. Additionally, the C_600_ residue of R-FPUF is only 2.3 wt.%, which is lower than that of P-FPUF (5.2 wt.%). The polyphosphoric acids and their derivatives result from the degradation of DDP and play a catalytic role in the dehydration and carbon-forming reactions that lead to the formation of stable chars [31,32,60,61].

As can be seen in Table 3 and Figure 8, the incorporation of EG leads to slight changes in T5, T_max1_, and RT_max1_. The T_max2_ values of P-RPUFs decrease consistently with the incorporation of EG. The remarkable decrease in T_max2_ for P-RPUF/15EG can be related to the decomposition process of EG [44,45,46,49]. As expected, the incorporation of EG leads to a decrease in RT_max2_ and an increase in C_600_. These observations can be attributed to the carbonaceous intumescent shield formed from the expansion of EG, which provides a layer of low thermal conductivity. The C_600_ value of P-RPUF/15EG is 12.2 wt.%, which is significantly higher than their counterparts without EG. The high char yield and low RT_max2_ often imply improved flame retardancy.

### 3.6. Analysis of Gas Phase

**Figure 9:**
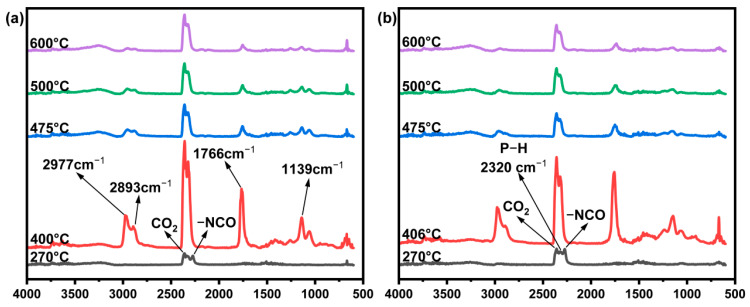
TG-IR spectra of the gaseous phase in the thermal degradation for R-FPUF (**a**) and P-FPUF (**b**) at different temperature.

To study the flame-retardant mechanism, the gaseous products formed during the thermal degradation process of the prepared foams were investigated using TG-IR. Figure 9 shows the TG-IR spectra acquired at different temperatures. The characteristic absorption bands of –NCO (2270 cm^−1^) and carbon dioxide (CO_2_, 2356 cm^−1^) are observed at T_5%_, indicating the degradation of the hard segments during the heating process [4,7,60]. When the temperature increases to T_max2_, strong characteristic signals for –CH_2_ and –CH_3_ (2976, 2932, and 2883 cm^−1^), C=O (1730 cm^−1^), and C–O (1145 cm^−1^) emerge due to the thermal cleavage of the soft segments [63,64]. It can be noted that a new absorption band at 2320 cm^−1^ attributed to the P–H stretching vibration [41,47] appears in the TG-IR spectrum of P-FPUF at 270 °C.

**Figure 10:**
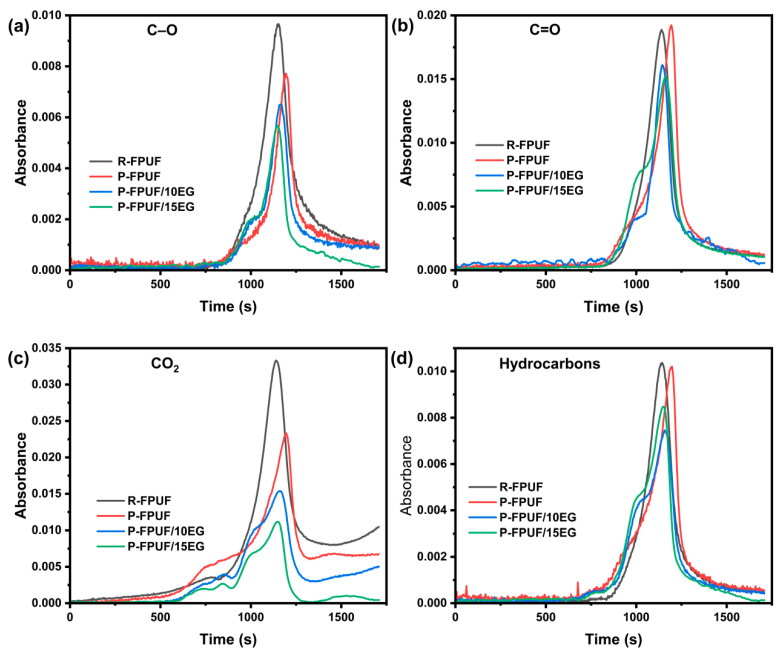
Intensities of absorption peaks for C─O (**a**), C=O (**b**), CO_2_ (**c**) and hydrocarbons (**d**) of R-FPUF, P-FPUF, P-FPUF/10EG, and P-FPUF/15EG.

To further investigate the change in the degradation products, the time-dependent evolution of CO_2_, hydrocarbons, C=O, and C–O signals were monitored. The obtained results are presented in Figure 10. As expected, the incorporation of phosphorus decreased the absorbance intensity of flammable organic gases and CO_2_, indicating that phosphorus provides great charring ability, which is consistent with the CC and TGA results mentioned above. As predicted, the incorporation of EG further decreased the absorbance intensity of flammable organic gases and CO_2_, confirming that the thick carbonaceous shield resulting from the expansion of EG traps the gases and provides great charring ability.

### 3.7. Analysis of Condensed Phase

**Figure 11:**
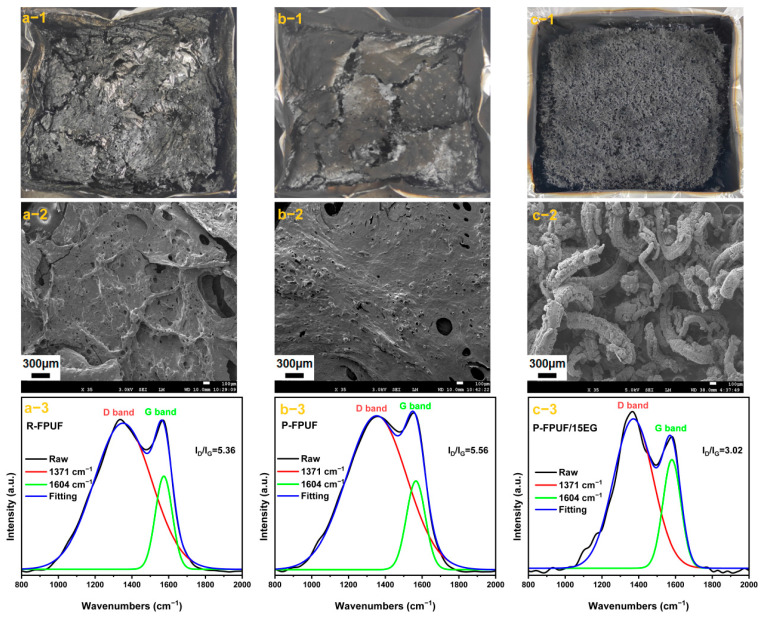
Morphology of the char residues and Raman spectroscopy of R-FPUF (**a–1**~**a–3**), P-FPUF (**b–1**~**b–3**), and P-FPUF/15EG (**c–1**~**c–3**).

The digital photos and SEM images for the char residues of the FPUFs with EG after the cone calorimetry tests are shown in Figure 11. The char residue of R-FPUF (Figure 11a–1) is very thin and fragmented. Some regions of the aluminum substrate are exposed because of the low char residue. Compared to the low char yield of R-FPUF, it was found that the addition of phosphorus obviously increases the char yield of P-FPUF, which indicates more pyrolysis products were turned into char. The surface of the char residue of P-FPUF is relatively smooth but presents several big cracks, indicating that the condensed-phase flame-retardant phosphorus still cannot provide a complete protection layer, leading to its unsatisfactory performance in the CC test, as also seen in previous studies [60]. As it can be seen in Figure 11c–1, after incorporating EG, the P-FPUF/15EG sample exhibits carbon layers in the shape of “worms”, revealing the protective effect during combustion, which was consistent with the cone result.

The carbon structure of the char was further analyzed by Raman spectroscopy. The D peak at about 1371 cm^−1^ corresponds to a defect peak of graphite, and the other G peak at 1604 cm^−1^ represents the regularity of graphite [65,66,67]. The intensity ratio of G band to D band (ID/IG) is used to characterize the graphitization degree. As can be seen in Figure 11a–3 and Figure 11b–3, the values of ID/IG for R-FPUF (5.36) and P-FPUF (5.56) char residue are very close, indicating that the degree of graphitization is not improved by the introduction of phosphorus. Obviously, with the addition of EG, the value of ID/IG of the P-FPUF/15EG sample was decreased to 3.02 (Figure 11c–3). The highly graphitized char layer has high strength and thermal stability, and it can effectively hinder the transfer of heat and flammable gases. The results are consistent with the observed results of char residue morphology and the CC test.

**Figure 12:**
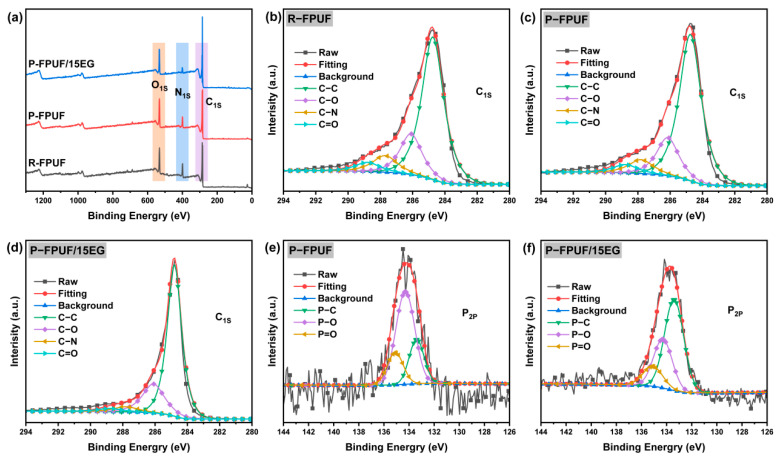
The XPS survey spectra of char residue for R-FPUF, P-FPUF, and P-FPUF/15EG (**a**), high-resolution XPS spectra of C1s for the R-FPUF (**b**), P-FPUF (**c**), and P-FPUF/15EG (**d**) char samples, high-resolution XPS spectra of P2p for the P-FPUF (**e**), and P-FPUF/15EG (**f**) char sam-ples.

The X-ray photoelectron spectroscopy (XPS) (Manufacturer, city, state abbreviation, country) spectra of the char residues for R-FPUF, P-FPUF, and P-FPUF/15EG samples obtained from the cone test are shown in Figure 12. They reveal that C, N, and O are the main elements remaining in the char residues of the three groups. For the control R-FPUF sample, the char residues consisted of C, O, and N, because no flame retardants were involved. The concentrations of C, O, and N in the char residues of the R-FPUF and P-FPUF samples are similar. The high-resolution XPS spectra of C1s and P2p for the R-FPUF, P-FPUF, and P-FPUF/15EG char samples are shown in Figure 12b–f. The C1s spectra of the R-FPUF and P-FPUF char residues shown in Figure 12b,c were similar and can be fitted to four characteristic peaks, namely C–C at 284.8 eV, C–O at 286.4 eV, C–N at 287.8 eV, and O–C=O at 288.5 eV [66]. Meanwhile, a peak is detected at 134 eV (P2p) [66,67] for the P-FPUF char residue sample, indicating that some phosphorus remains in the char residue. Approximately 12.4 wt.% of the phosphorus remained in the P-FPUF char residue based on the comparison of the phosphorus content in the char residue (1.4 wt.%) and the corresponding original foam (0.72 wt.%), indicating that phosphorus mainly acted as a gas-phase flame retardant.

Remarkably, as can be seen in Figure 12c, although the C1s spectrum of the P-FPUF/15EG char residue can also be fitted to the four characteristic peaks of C–C, C–O, C–N, and O–C=O, the concentration of each bond was significantly lower than that of the R-FPUF and P-FPUF char samples, indicating a high degree of graphitization, similar to the Raman results. The P2p spectra of the P-FPUF char sample presented in Figure 12e,f were fitted to three characteristic peaks of 133.4, 134.3, and 135.1 eV, corresponding to the P–C, P–O, and P=O groups, respectively [67]. The concentration of phosphorus in the P-FPUF/15E char sample was higher than that of the P-FPUF sample (see Table 4). By calculation, approximately 75.4% of the phosphorus remained in the P-FPUF/15EG char residue. A high phosphorus content improves the oxidation resistance and mechanical strength of the char, which is beneficial for the formation of a high-quality char layer [17].

### 3.8. Flame-Retardant Mechanism

**Figure 13:**
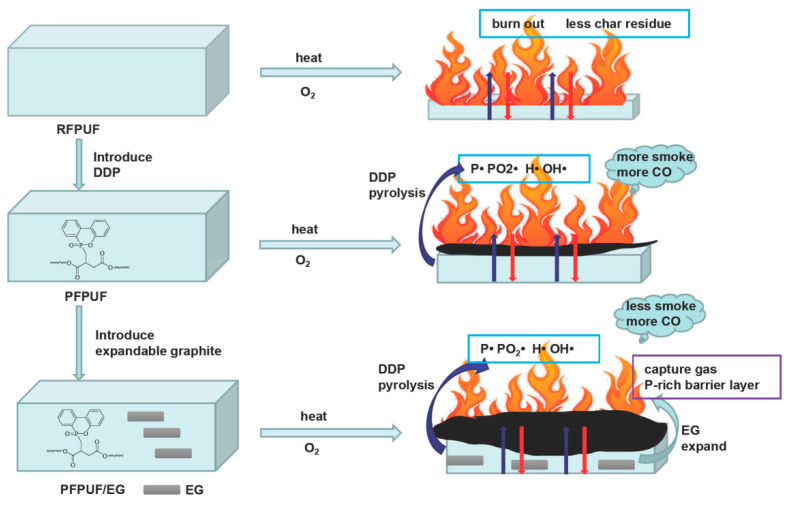
Schematic illustration of possible flame-retardant mechanism of phosphorus and EG in P-FPUFs.

Combining the above analyses and referring to the literature, it is concluded that for the control R-FPUF sample with no flame retardants, the heat transfers quickly, resulting in the combustion of the polymer. The introduction of phosphorus increased the yield of char residue owing to a condensed-phase flame-retardant mechanism. Meanwhile, the gas-phase flame-retardant mechanism resulting from volatile phosphorus-containing compounds in the gas phase improved the LOI value but produced a large amount of smoke during combustion. With the loading of EG, the resulting phosphorus-rich intumescent carbonization layers acted as physical protective layers as well as gas scavengers. The formation of a dense network of highly graphitized char layers isolated the external oxygen, flammable gas, and heat from the internal matrix and effectively suppressed the release of heat and smoke. Overall, the combination of phosphorus and EG depressed combustion and led to lower THR and PHRR values, as well as a low smoke release. Figure 13 shows the flame-retardant mechanism of phosphorus and EG in the P-FPUFs.

**Figure 14:**
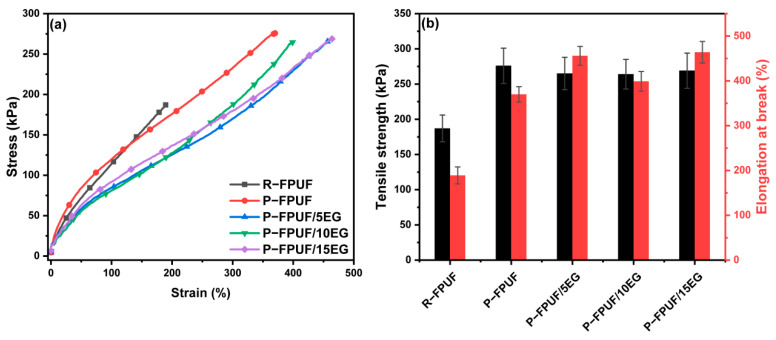
Mechanical properties of pure FPUF (**a**) and flame-retardant FPUFs (**b**).

The effect of PPE and/or EG on the mechanical properties of R-FPUFs and P-FPUFs was evaluated by measuring the tensile strength and elongation at break (Figure 14). As presented in Figure 14, the R-FPUF sample has a common tensile strength and elongation at break of about 190 KPa and 180%, respectively. Remarkably, higher tensile strength above 250 kPa and elongation at break more than 300% are observed for all P-FPUF samples, which can be attributed to the strong interactions between the soft and hard segments of the FRPE chains with the introduction of 9,10-Dihydro-10[2,3-di(hydroxycarbonyl)propyl]10-phosphaphenanthrene-10-oxide (DDP) [60]. These good mechanical properties make P-FPUF highly attractive for use in practical application.

## 4. Conclusions

Inherently flame-retardant and smoke-suppressant FPUFs were obtained via the reaction of toluene diisocyanate and a reactive phosphorus-containing flame-retardant polyester diol made from 9,10-dihydro-10-[2,3-di(hydroxycarbonyl)propyl]-10-phospha-Phenanthrene-10-oxide, adipic acid, ethylene glycol, and 1,4-butanediol. Compared to the regular FPUF with no flame retardants, the introduction of phosphorus improves the LOI value and char residue yield of P-FPUFs but produces a large amount of smoke during combustion. With further loading of EG, the resultant P-FPUF/EG foams exhibited good mechanical properties as well as flame-retardant, anti-dripping, and smoke suppression performances. This superior flame-retardant performance can be explained by the combination of the gas-phase flame-retardant characteristics of phosphorus-containing volatile compounds and the condensed-phase flame-retardant behaviors of EG and phosphorus. This superior performance and the simplicity of this strategy make it highly attractive for use in preparing new flame-retardant FPUFs.

## Figures and Tables

**Table 1:** The vertical burning test results of R-FPUF and flame-retardant FPUFs.

Sample	Pass/No (TB117)	Dripping (Yes/No)	Sample	Pass/No (TB117)	Dripping (Yes/No)
R-FPUF	No	Yes	R-FPUF/15EG	Pass	No
P-FPUF	No	Yes	P-FPUF/5EG	Pass	No
R-FPUF/5EG	No	Yes	P-FPUF/10EG	Pass	No
R-FPUF/10EG	No	No	P-FPUF/15EG	Pass	No

**Table 2:** Characteristic parameters of CC tests of FPUFs.

Sample	TTI (S)	PHRR (kW/m^2^)	TPHRR (s)	FIGRA	THR (MJ/m^2^)	PSPR (m^2^/s)	TSP (m^2^)	AEHC (MJ/Kg)	COY (kg/kg)	CY (%)
R-RPUF	9	640	61	10.5	32.0	0.036	1.22	22.6	0.023	2.5
P-RPUF	12	521	70	7.4	26.8	0.104	3.25	26.4	0.108	6.4
P-RPUF/10EG	19	185	40	4.6	24.7	0.031	1.71	30.3	0.101	18.0
P-RPUF/15EG	7	90	23	3.9	16.0	0.009	0.54	28.5	0.207	28.6

**Table 3:** TGA data of FPUFs at a heating rate of 20 °C/min in N_2_.

Sample	T_5_ (°C)	T_max1_ (°C)	RT_max1_ (%/min)	T_max2_ (°C)	RT_max2_ (%/min)	C_wt_600 (%)
R-FPUF	251	280	7.9	400	25.7	2.3
P-FPUF	254	277	9.0	419	25.2	5.2
P-FPUF/10EG	248	272	8.9	408	21.9	9.6
P-FPUF/15EG	246	273	8.5	403	21.2	12.2

**Table 4:** Elemental composition and content of residual char.

Sample	C (wt.%)	N (wt.%)	O (wt.%)	P (wt.%)
R-FPUF	67.3	13.3	19.4	0
P-FPUF	69.9	9.9	19.9	1.4
P-FPUF/15EG	81.4	4.5	13.8	1.9

## Data Availability

Not applicable.

## Supplementary Materials

The following supporting information can be downloaded at: https://www.mdpi.com/article/10.3390/polym15051284/s1, Figure S1: The molecular weight distribution curve of FRPE; Figure S2: SEM image for cross sections of P-FPUF/15EG. Table S1: The formulae, densities and LOI values of flame-retardant FPUF samples; Table S2: The vertical burning test results of FPUFs.
