# Supplementary material for: Flame-Retardant and Smoke-Suppressant Flexible Polyurethane Foams Based on Phosphorus-Containing Polyester Diols and Expandable Graphite
Source: Polymers (Basel). 2023 Mar 3;15(5):1284. doi: 10.3390/polym15051284 (PMC10006967; doi:10.3390/polym15051284)
Supplement: Supplementary file 1 [file polymers-15-01284-s001.zip › polymers-2166492-supplementary.pdf]

polymers

MDPI

Article

Flame-Retardant and Smoke-Suppressant Flexible Polyurethane Foams Based on Phosphorus-Containing Polyester Diols and Expandable Graphite

Hongkun Wang, Qiang Liu *, Hui Li *, Hao Zhang and Shouke Yan *

Key Laboratory of Rubber-Plastics, Ministry of Education/Shandong Provincial Key Laboratory of Rub-ber-Plastics, Qingdao University of science & technology, Qingdao 266042, China
* Correspondence: liuqiang@qust.edu.cn (Q.L.); fs408@qust.edu.cn (H.L.); skyan@qust.edu.cn (S.K.Y.)

Contents

1.Materials
2.Measurements
3.Table S1. The formulae, densities and LOI values of flame-retardant FPUF samples.
4.Table S2.The vertical burning test of pure FPUFs and flame-retardant FPUFs.
5.Figure S1. The molecular weight distribution curve of PPE.
6.Figure S1. SEM image for cross sections of P-FPUF/15 EG

1. Materials

9,10-dihydro-10-[2,3-di(hydroxycarbonyl)propyl]-10-phosphaphenanthrene-10-oxide (DDP) was provided by Zhengzhou Alpha Chemical Co., Ltd., Zhengzhou, China. 1,4-butylene glycol (1,4−BDO) and ethylene glycol (EGO) were purchased by Sinopec Beijing Yanshan Petrochemical Co., Ltd., Beijing, China. Tetrabutyl titanate (TBT) and Adipic acid (AA) were supplied by Tianjin Fuchen Chemical Reagent Factory, Tianjin, China. Bis(2-dimethylaminoethyl) Ether was supplied by Energy Chemical, Shanghai, China. Triethylenediamine (A−33) were received from Jiangsu Maysta Chemical Co., Ltd., Nanjing, China. T9 Tin(II)2-ethylhexanoate was obtained from Energy Chemical, Shanghai, China. Toluene diisocynate (TDI, technical pure grade) was supplied from INOV New Material Co., Ltd., Zibo, China. Distilled water was used as reaction initiator for blowing agent of carbon dioxide. Silicone stabilizer (S−232) was used as surfactant and supplied by MENHOVER, Shanghai, China. Regular polyester diol (RPE, synthesized from AA, 1,4-BDO, EG, molar ratio of the two dihydric alcohols is 1, hydroxyl value is 56 mg KOH/g) was supplied from INOV New Material Co., Ltd., Zibo, China, the molar ratio of the two dihydric alcohols is 1.

2. Measurements

Limiting oxygen index (LOI) value was carried out according to standard method ISO 4589−1: 1996 with an HC−2C oxygen index instrument (TESTech Instrument (Suzhou) Technologies, Suzhou, China). The sample of the FPUFs were 150 × 10 × 10 mm^3.

Vertical burning tests of FPUF samples were conducted according to California technical bulletin 117 (TB 117−2000) and the specimen dimension was 12 × 3× 1/2 in.

The densities of the FPUFs were measured according to ISO 845:2006. The size of the specimen was no less than 100 cm^3, and the average density was the average of five specimens.

Thermogravimetric analysis (TGA) was performed using a Netzsch 209 F1 thermal analyzer (Germany Netzsch Instrument Manufacturing Co., Ltd.,Bavaria, Geramany). The sample (3−5 mg) was heated from room temperature to 600 °C at a heating rate of 20 °C/min under a nitrogen atmosphere.

The mechanical properties of the samples were determined by tensile measurements with a ZBC1400−2 testing machine (Company Files, Ningbo, China) according to standard method ISO 1798:2008.

Thermogravimetric analysis/infrared spectrometry (TG−IR) was performed using a VERTEX 70 FTIR (Bruker Corporation, Biblica, America) spectrophotometer and a TG209F1 (Germany Netzsch Instrument Manufacturing Co., Ltd.,Bavaria, Geramany) thermal-analyzer instrument to analysize the gaseous products of samples during the decomposition. About 3 mg of the sample was heated from room temperature to 600 °C a heating rate of 20 °C under a the nitrogen flow.

Cone calorimeter (CC) tests were performed with a cone calorimeter (Phinix Instrument, Xuzhou, China) at a heat flux of 25 kW/m^2 according to standard method ISO 5660−1. Each specimen with dimensions of 100 × 100 × 25 mm^3. The separation distance between the cone heater and the specimen is 25 mm.

Scanning electron microscopy(SEM) images were characterized using a JEOL−JSM−7500F spectrometer (JEOL, Kyushu, Japan). Cross-sectional morphologies were recorded after gold coating surface treatment under a high vacuum at a voltage of 20 kV.

The X-ray photoelectron spectroscopy (XPS) of the char residue and coatings samples were obtained by an ESCALAB250 (Thermo VG, Shanghai, China) spectrometer with an Al Ka excitation radiation . C1s spectra was shifted to standard positions of 284.8 eV. The XPSPEAK software was used for fitting.

Raman spectra were acquired from a HORIBA HR Evolution Raman spectrometer (Horiba, Shanghai, China) using a wave number range of 500–2000 cm^{-1} with the 514 nm argon laser.

The molecular weight and its distribution of FRPE was measured by gel permeation chromatography (GPC) using a Waters 1515 HPLC system (Waters, Massachusetts, America) equipped with Ultrastyragel® columns and differential refractometer detector. Tetrahydrofuran (THF) was used as eluent at a flow rate of 1.0 mL/min at 25°C. The molecular weight of samples was calculated from the polystyrene standards with narrow polydispersity.

3. Table S1.

Table S1. The formulae, densities and LOI values of flame-retardant FPUF samples.

Sample	Y−173	FRPE	S−232	H_2O	A−1	A−33	T−9	EG	TDI	LOI	Density
				Unit: g						(%)	(kg/m^3)
R-FPUF	100	0	1	2	0.1	0.2	0.2	0	28.0	18.2 ± 0.5	45.6
R-FPUF/5EG	100	0	1	2	0.1	0.2	0.2	5	28.0	21.6 ± 0.4	56.3
R-FPUF/10EG	100	0	1	2	0.1	0.2	0.2	10	28.0	24.9 ± 0.7	62.1
R-FPUF/15EG	100	0	1	2	0.1	0.2	0.25	15	28.0	27.3 ± 0.1	75.8
P-FPUF	0	100	1	2	0.1	0.2	0.2	0	28.0	21.4 ± 0.2	53.7
P-FPUF/5EG	0	100	1	2	0.1	0.2	0.2	5	28.0	22.5 ± 0.4	60.4
P-FPUF/10EG	0	100	1	2	0.1	0.2	0.2	10	28.0	28.1 ± 0.4	66.2
P-FPUF/15EG	0	100	1	2	0.1	0.2	0.25	15	28.0	29.2 ± 0.6	91.2

4. Table S2.

Table S2. The vertical burning test results of FPUFs.

Sample	After Flame Time (s)		After Glow Time (s)	Char Length (mm)		Test Results (Pass or Fail)
	Average	Maximum	Average	Average	Maximum	
Requirements for pass	≤5.0	≤15.0	≤15.0	≤152.4	≤203.2	Pass
R-FPUF	45	45	0	No	Burn out	No
P-FPUF	40	40	0	No	Burn out	No
R-FPUF/5EG	50	50	0	No	Burn out	No
R-FPUF/10EG	18	20	0	45	45	No
R-FPUF/15EG	2	2	0	15	15	Pass
P-FPUF/5EG	5	5	0	25	25	Pass
P-FPUF/10EG	0	0	0	20	20	Pass
P-FPUF/15EG	0	0	0	10	11	Pass

5. Figure S1

Peak Results

Signal name	Peak start RT (min)	RT (min)	Peak end RT (min)	Area	Area %
RID1A	11.056	13.130	15.918	257388.5408	100.00

Molecular Weight Averages

Peak #	RT (min)	Mp (g/mol)	Mn (g/mol)	Mw (g/mol)	Mz (g/mol)	Mz+1 (g/mol)	PD
1	13.130	2492	1562	2446	3375	4218	1.565941

Chromatogram

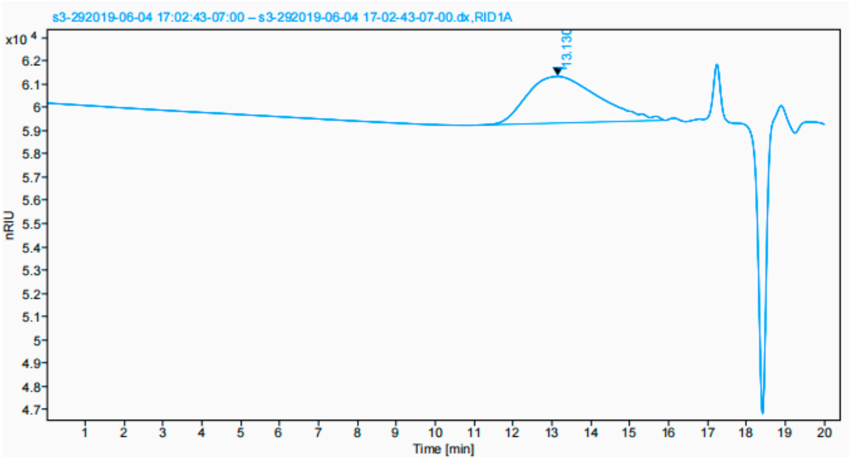

Figure S1. The molecular weight distribution curve of FRPE.

6. Figure S2.

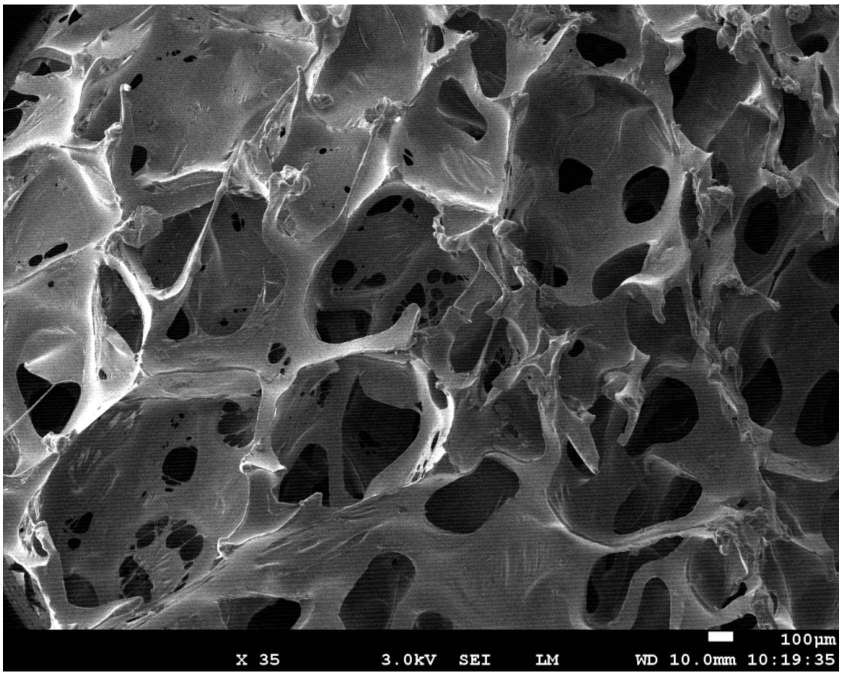

Figure S2. SEM image for cross sections of P-FPUF/15EG.
